# Addressing the unmet need in NSCLC progression with advances in second-line therapeutics

**DOI:** 10.37349/etat.2024.00277

**Published:** 2024-11-01

**Authors:** Kinsley Wang, Alexis Leyba, Robert Hsu

**Affiliations:** IRCCS Istituto Romagnolo per lo Studio dei Tumori (IRST) “Dino Amadori”, Italy; ^1^Department of Medicine, University of Arizona College of Medicine, Phoenix, AZ 85004, USA; ^2^Department of Medicine, University of Southern California Keck School of Medicine, Los Angeles, CA 90033, USA; ^3^Department of Medicine, Division of Medical Oncology, University of Southern California Norris Comprehensive Cancer Center, University of Southern California Keck School of Medicine, Los Angeles, CA 90033, USA

**Keywords:** Non-small cell lung cancer (NSCLC), antibody-drug conjugates (ADCs), second-line therapy, immunotherapy, cancer progression

## Abstract

Lung cancer is the leading cause of cancer mortality globally, with non-small cell lung cancer (NSCLC) accounting for 85% of cases. Despite advancements in first-line treatments such as immunotherapy and targeted therapies, resistance to these treatments is common, creating a significant unmet need for effective second-line therapies. This review evaluates current and emerging second-line therapeutic options for advanced or metastatic NSCLC, focusing on their efficacy and potential to improve patient outcomes. Anti-angiogenic drugs like ramucirumab combined with chemotherapy, particularly docetaxel, have shown moderate success. Antibody-drug conjugates (ADCs) targeting specific tumor antigens offer a promising avenue for targeted therapy, while chimeric antigen receptor (CAR)-T cell therapy and T-cell receptor therapy leverage the patient’s immune system to combat cancer more effectively. mRNA vaccines, although in early stages, show potential for inducing robust immune responses against cancer-specific antigens. Building on this foundation, recent advancements in molecular testing and the exploration of the tumor microenvironment are opening new therapeutic avenues, further enhancing the potential for personalized second-line treatments in NSCLC. While ADCs and bispecific antibodies are gaining traction, more precise biomarkers are needed to optimize treatment response. Regular monitoring through techniques like liquid biopsies allows real-time tracking of mutations such as EGFR T790M, enabling timely therapeutic adjustments. Additionally, the role of neutrophils and macrophages in the tumor microenvironment is increasingly being recognized as a potential therapeutic avenue, with Smad3 emerging as a key target. Further research into drug sequencing, toxicity management, and biomarker development remains crucial to improving NSCLC treatment outcomes.

## Introduction

Lung cancer is the leading cause of cancer mortality worldwide, with non-small cell lung cancer (NSCLC) accounting for approximately 85% of all cases [1]. The two most common subtypes are adenocarcinoma, making up 40–50% NSCLC cases, and squamous cell carcinoma, accounting for 25–30% [1]. The prognosis for NSCLC is generally poor, particularly when diagnosed at an advanced/metastatic stage without targetable mutations. Targetable mutations only occur in 30–60% of NSCLC patients, though this proportion varies based on geographic and demographic factors. The most common mutations include EGFR (10–20%), KRAS (10–30%), ALK (1–5%), ROS1 (1–5%), RET (1–5%), and MET exon 14 skipping mutations (1–5%). These frequencies are population-dependent, with mutations like EGFR being more prevalent in Asian populations. Previously, patients without targetable mutations received platinum-doublet chemotherapy. Now, due to advances in immune checkpoint inhibitors (ICIs), almost all patients receive first-line immunotherapy or immuno-chemotherapy [2–5]. However, resistance to targeted therapies, immunotherapy, and/or chemotherapy is common, so further research must be done for effective second-line treatments, necessitating a review of current and in-development second-line treatment options and their efficacy in treating NSCLC.

First line treatments have been reviewed in detail elsewhere [1, 6]. Briefly, they typically include surgical resection, radiation, targeted therapies, immunotherapies, and chemotherapy, chosen based on the tumor’s genetic profile and patient’s overall health. Early-stage NSCLC (stages I, II, and IIIA) is recommended surgical resection or radiation, with stages II and IIIA also undergoing adjuvant immunotherapy, targeted therapy, or platinum-based chemotherapy. Unresectable tumors are treated with chemoradiation followed by either immunotherapy or targeted therapy.

**Figure 1:**
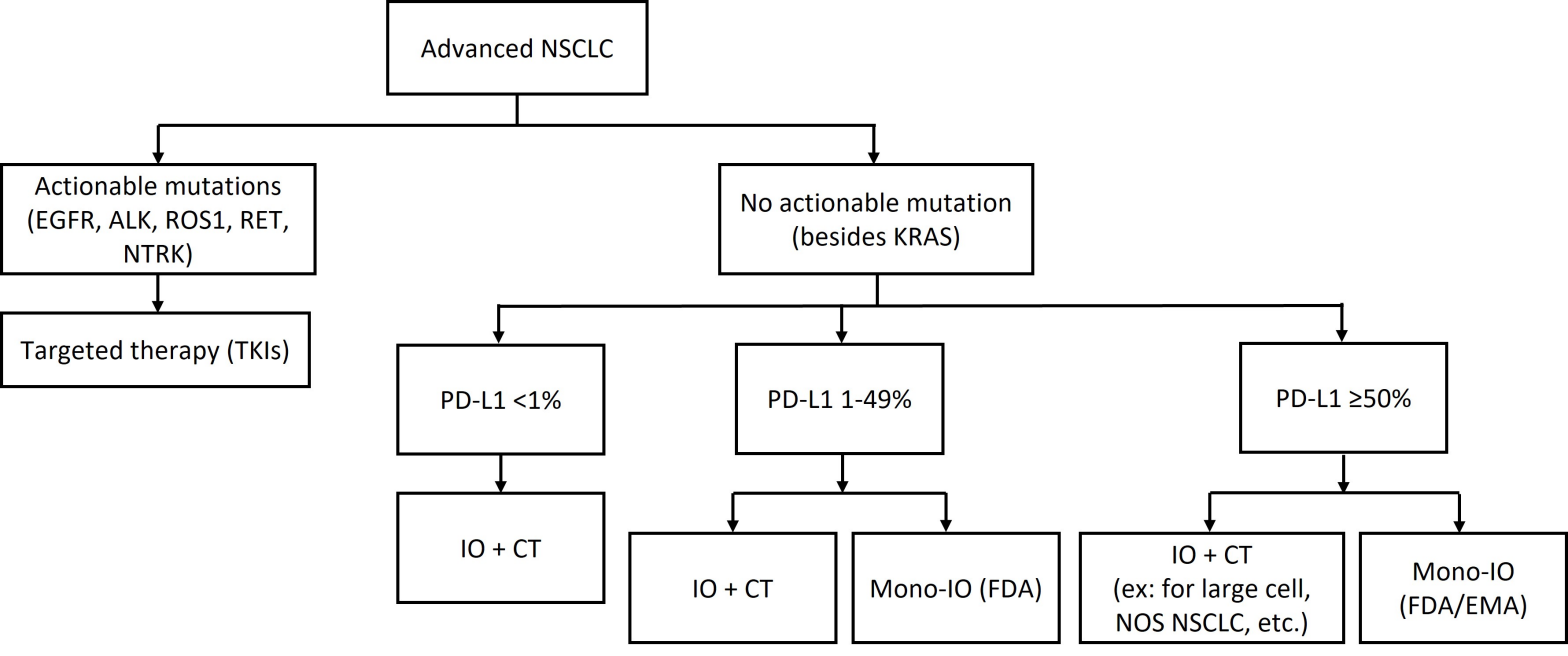
First-line treatments for advanced NSCLC. NSCLC: non-small cell lung cancer; IO: immunotherapy; CT: chemotherapy; Mono-IO: immunotherapy monotherapy; ex: atezolizumab monotherapy, pembrolizumab monotherapy

Later-stage NSCLC treatment depends on whether actionable mutations are present, as seen in the therapeutic algorithm for first-line treatments in Figure 1. Targeted therapies such as EGFR tyrosinase kinase inhibitor (TKI) (e.g., osimertinib) improve progression-free survival (PFS), showing a 54% relative improvement, and overall survival (OS), with an OS benefit of 6.8 months [7, 8]. For patients without driver mutations, treatment involves immunotherapy and chemotherapy. In PD-L1 positive [tumor proportion score (TPS) ≥ 50%] tumors, single-agent immunotherapy, such as pembrolizumab, improves PFS and OS, with 31.9% reaching 5-year survival, compared to 16.3% for chemotherapy [9, 10]. Other ICIs such as atezolizumab and cemiplimab have also been highly effective [5, 11]. PD-L1 negative (< 1%) and PD-L1 (1–49%) tumors are typically treated with combination immunotherapy and chemotherapy. Trials such as KEYNOTE-189 and KEYNOTE-407 have demonstrated significant survival benefits with pembrolizumab plus chemotherapy combinations in non-squamous (NSQ) and squamous (SQ) NSCLC, respectively [2, 3]. Doublet immunotherapy trials like the CHECKMATE-9LA trial, which tested nivolumab plus ipilimumab plus platinum-doublet chemotherapy vs. chemotherapy, demonstrated improved OS of 15.8 months [4]. Similarly, POSEIDON trial evaluated tremelimumab plus durvalumab plus chemotherapy, durvalumab plus chemotherapy, and chemotherapy alone, demonstrating that a limited course of tremelimumab added to durvalumab plus chemotherapy significantly improved PFS and OS compared to chemotherapy, without meaningful additional tolerability burden.

Despite progress with first-line immunotherapy agents, both primary resistance, where tumor evaluation after < 6 weeks is progressive disease (PD) or stable disease (SD), and secondary resistance, where tumor response to treatment reached complete response (CR), partial response (PR), or SD ≥ 6 months and then PD, commonly occur. For immunotherapy alone, 21–27% of patients have primary resistance and 52–57% have secondary; for combined immunotherapy-chemotherapy, primary resistance was 10%, which translates to five-year disease-free survival rate of 7.5–10.8% [12, 13]. This underscores the need to develop more effective second-line therapy for after progression.

## Treatments following progression

**Figure 2:**
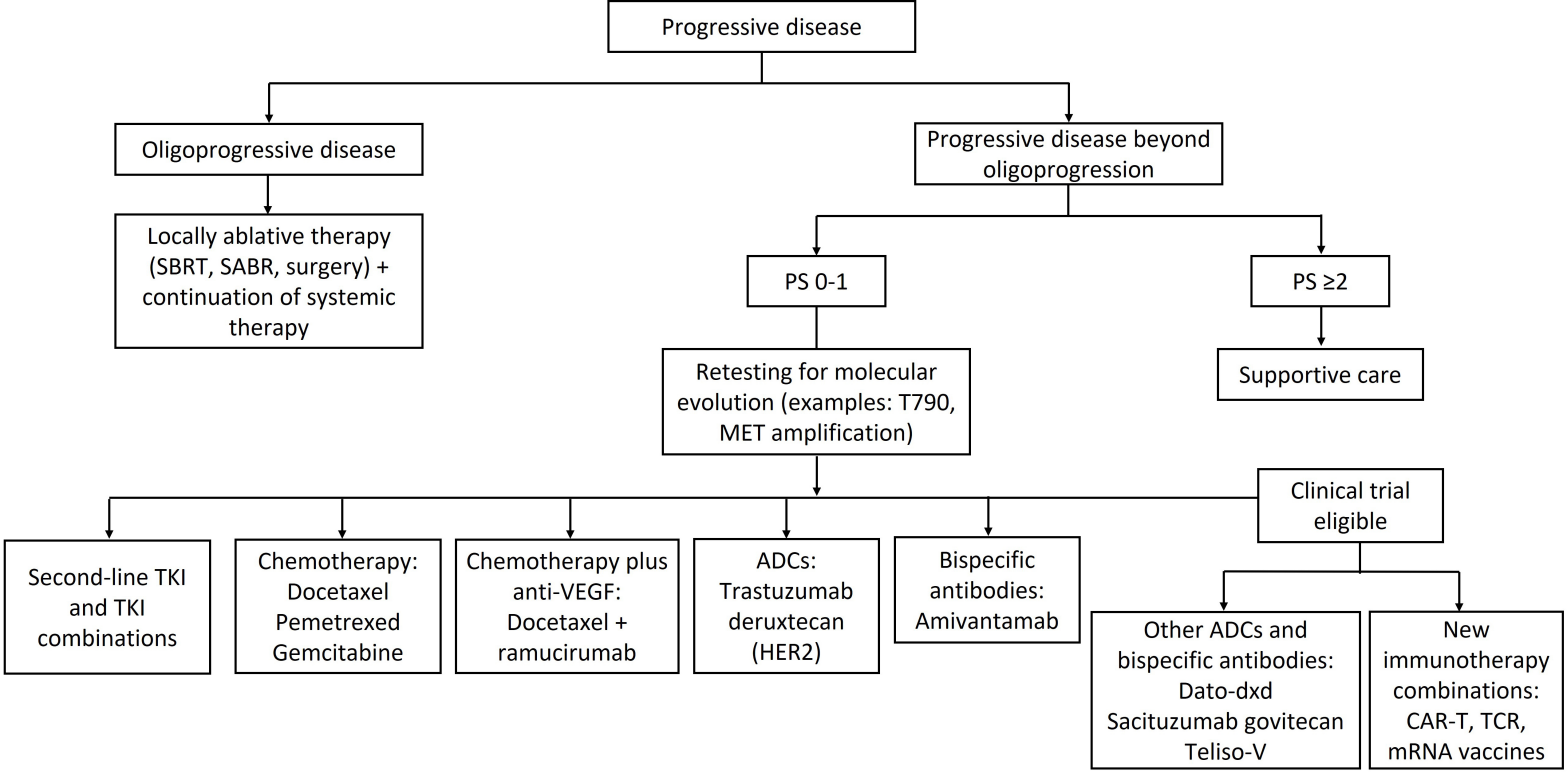
Second-line treatments for metastatic NSCLC following progression. SBRT: stereotactic body radiation therapy; TKI: tyrosinase kinase inhibitor; ADC: antibody-drug conjugate; CAR-T: chimeric antigen receptor T cell therapy; TCR: T-cell receptor therapy; NSCLC: non-small cell lung cancer; PS: performance status

Many new treatments for PD beyond oligoprogression have been developed, such as second-line TKIs, chemotherapy combinations, anti-angiogenic agents, antibody-drug conjugates (ADCs), bispecific antibodies, and new immunotherapy combinations (Figure 2). However, despite the availability of multiple treatment options, there have been a lack of options that provide durable response. NSCLC that has progressed after first-line treatment has poor prognosis, with response rates less than 10% to docetaxel and a median survival of 7–9 months from the start of second-line therapy [14]. There is unmet need of effective treatment options in advanced or metastatic NSCLC following progression.

### Radiation and surgery in oligoprogressive disease

Oligoprogression is generally characterized by a limited number of metastatic lesions (typically 3–5), while the remaining disease remains stable or controlled [15]. Oligoprogression allows local therapies, such as surgery and radiotherapy, to be applied to progressing sites, enabling continued systemic treatment for the rest of the cancer. Growing evidence supports the use of locally ablative treatments, including radiotherapy and surgical interventions, at metastatic locations [16–18]. However, the absence of prospective data leaves the optimal treatment strategy uncertain.

Advances in radiotherapy techniques, such as stereotactic body radiation therapy (SBRT) and SABR, which minimize damage to healthy tissues, have made less invasive local treatments more appealing [19]. A randomized trial compared standard of care (SOC) with or without SBRT in NSCLC and breast cancer patients with oligoprogressive metastases (progression ≤ 5 individual lesions) who had received at least one line of systematic therapy. SBRT significantly extended median PFS (mPFS) in NSCLC patients: PFS was 10.0 months for SBRT cohort and 2.2 months for SOC cohort, though so significant difference was observed in breast cancer. 16% of patients experienced grade 2 or higher toxicities associated with SBRT, including pneumonitis [20].

Surgical removal of oligoprogressive lesions (metastasectomy) is another effective local treatment, particularly for isolated metastases in organs like the lung, liver, and brain [21]. In addition to removing resistant lesions, surgery provides tissue samples to study resistance mechanisms. Although not specific to oligoprogression, a phase 3 randomized controlled trial assessed the effect of adding surgical resection to palliative whole brain radiotherapy in NSCLC patients who had a single brain metastasis [22]. Patients that underwent resection had a significantly longer survival (40 weeks vs. 15 weeks). A meta-analysis focusing on patients with oligometastatic NSCLC found that nearly two-thirds of the included patients underwent surgery as the main treatment, and surgery was significantly associated with improved OS [20]. However, there was potential for selection bias that favored better outcomes in the surgical group, because patient selection for surgery depends on overall health, limited metastatic spread, and sufficient respiratory function.

## Anti-angiogenic agents

Chemotherapy, such as docetaxel, pemetrexed, or gemcitabine, has traditionally been the SOC for metastatic NSCLC patients who progress on first-line treatments [23]. Advances in anti-angiogenic drugs, such as nintedanib or ramucirumab, which inhibit tumor blood supply, have demonstrated clinical benefit when combined with chemotherapy [24, 25]. A phase 3 study, REVEL, evaluated ramucirumab plus docetaxel in patients that progressed on platinum-based chemotherapy [24]. PFS was 4.5 months for ramucirumab cohort vs. 3.0 months for docetaxel (HR 0.76, *P* < 0.0001); OS was 10.5 months vs. 9.1 months (HR 0.86, *P* = 0.023), respectively. Similar results were observed for docetaxel plus nintedanib [25]. Today’s second-line options have expanded to docetaxel, pemetrexed, or gemcitabine, or docetaxel in combination with nintedanib or ramucirumab.

Recently, anti-angiogenics have been combined with immunotherapy in second-line therapy. A phase 2 trial, Lung-MAPS1800A, showed ramucirumab plus pembrolizumab improved OS to 14.5 months compared to 11.6 months with SOC (HR 0.69, *P* = 0.05) in patients previously treated with immunotherapy and platinum-based chemotherapy [26]. Other trials are testing combinations of immunotherapy and anti-angiogenics. One recent example is CONTACT-01, a phase 3 trial which compared atezolizumab plus cabozantinib vs. docetaxel; PFS was 4.6 months vs. 4.0 months and OS was 10.7 months vs. 10.5 months (HR 0.88, *P* = 0.37), respectively, demonstrating a modest improvement [27]. Another ongoing phase 2 trial is investigating the combination of docetaxel, ramucirumab, and pembrolizumab after progression on chemotherapy and immunotherapy (*n* = 41) [28].

Interestingly, recent trials show improved outcomes with docetaxel compared to earlier studies. For instance, in 2023 study, docetaxel’s PFS was 4.5 months and OS was 11.3 months (*n* = 151), compared to a 2014 study where docetaxel’s PFS was 3.0 months and OS was 9.1 months (*n* = 625) [24, 29]. This effect could be attributed to chemotherapy’s effect on the immune system, by increasing the immunogenicity of malignant cells or by inhibiting the immune suppression of cancer tumors [30].

### Antibody-drug conjugates

ADCs are an emerging class of therapeutic agents with high specificity, limiting toxicity. ADCs are made from a monoclonal antibody targeting a tumor-specific receptor, a linker that controls drug release, and a cytotoxic payload, typically a tubulin inhibitor, DNA damage inducer, or immunomodulator [31]. The drug-antibody ratio (DAR) indicates the number of drug molecules attached per antibody. While higher DAR increases efficacy, it can also affect antibody stability and binding, influencing the therapeutic index [32]. DAR can be determined through high-performance liquid chromatography-ultraviolet spectroscopy [33].

**Figure 3:**
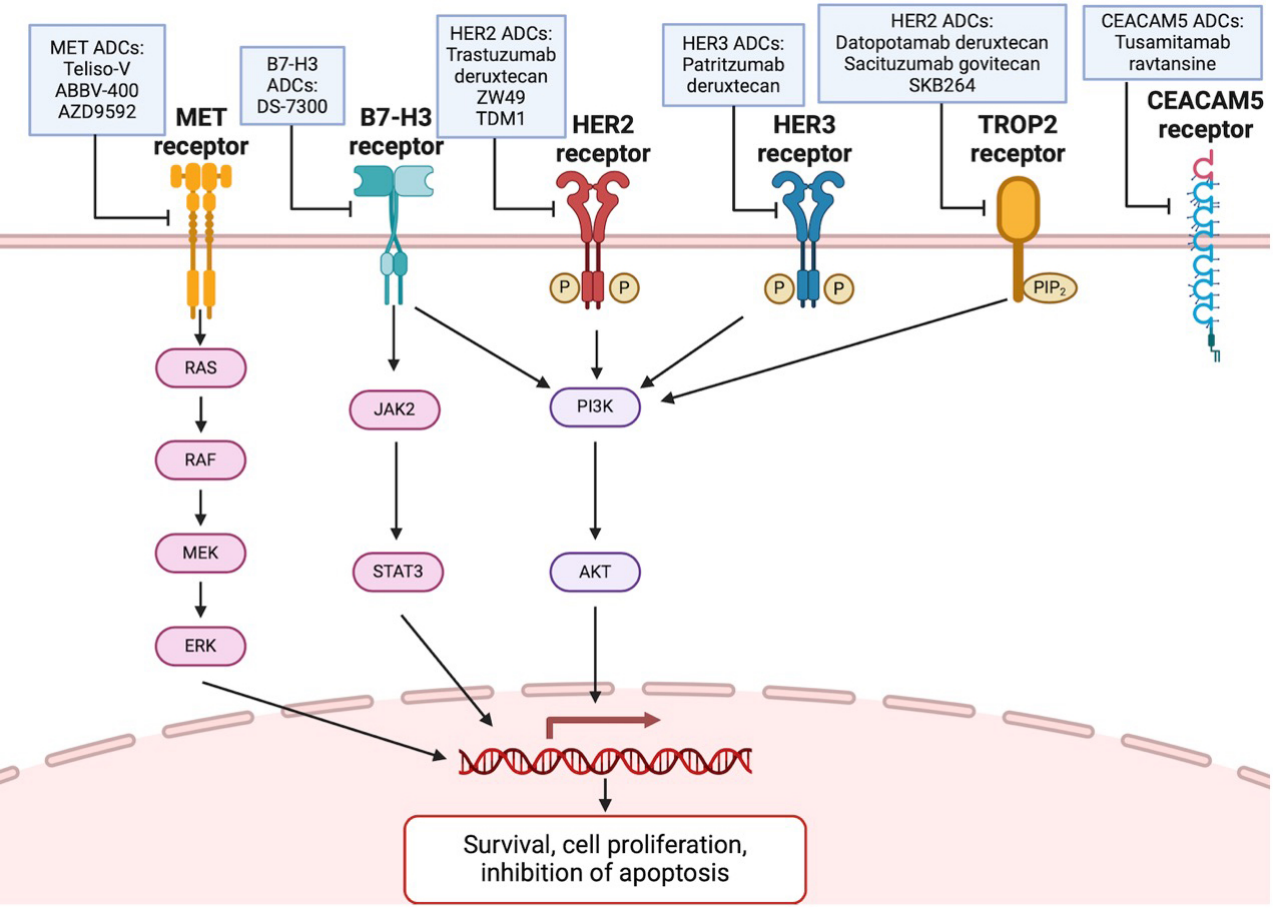
Signaling pathways of ADCs targeting MET, B7-H3, HER2, HER3, Trop-2, and CEACAM5. Created in BioRender. Wang, K. (2024) BioRender.com/a27s324. ADCs: antibody-drug conjugates; HER2: human epidermal growth factor receptor 2; TDM1: ado-trastuzumab emtansine; Trop-2: trophoblast cell-surface antigen 2; CEACAM5: cell adhesion molecule 5

The mechanism of action is antibody-mediated receptor binding to target cells, internalization of ADC through receptor-mediated endocytosis, and the lysosomal cleavage of ADC linker, leading to release of payload and cytotoxic action [34]. ADCs also have a bystander effect, where the internalized drug is released by cells. Adjacent tumor cells then take up the drug, thus increasing the killing efficacy of ADCs, particularly in heterogeneous tumors or homogeneous tumors with low target expression [35]. Many ADCs are in development targeting oncogenic signaling pathways and demonstrate promising clinical data (Table 1, Figure 3).

**Table 1:** Current clinical data on ADCs in NSCLC

**Target**	**Drug**	**Trial, phase**	**Outcomes**
*cHER2*	Trastuzumab deruxtecan (T-DXd)	DESTINY-Lung01 [36, 37] (6.4 mg/kg) *N* = 91 Phase 2	ORR 54.9% DOR 10.6 mo mPFS 8.2 mo mOS 18.6 mo DCR 92%
		DESTINY-Lung02 [38] (5.4 mg/kg vs. 6.4 mg/kg) *N* = 101 vs. 50 Phase 2	5.4 mg/kg vs. 6.4 mg/kg: ORR 53.8% vs. 42.9% DOR NE vs. 5.9 mo DCR 90.4% vs. 92.9%
	Ado-trastuzumab emtansine (TDM1)	JapicCTI-194620 [39] *N* = 22, HER2 exon-20 mutation Phase 2	ORR 38.1% DOR 3.5 mo mPFS 2.8 mo mOS 8.1 mo DCR 52.4%
		NCT02289833 [40] (IHC 2+ vs. 3+) *N* = 29 vs. 20 Phase 2	IHC 2+ vs. 3+: ORR NE vs. 20% mPFS 2.6 mo vs. 2.7 mo mOS 12.2 mo vs. 15.3 mo
	Zanidatamab zovodotin (ZW49)	NCT03821233 [41] Phase 1 Reported for multiple cancer types	ORR 28% DCR 72%
*HER3*	Patritzumab deruxtecan (HER3-DXd)	NCT03260491 [42, 43] (EGFR-WT vs. EGFRm) *N* = 47 vs. 57 Phase 1	EGFR-WT vs. EGFRm: ORR N/A vs. 39% DOR 5.7 mo vs. 6.4 mo DCR 28% vs. 72% mPFS 5.4 mo vs. 8.2 mo
		HERTHENA-Lung01 [44] *N* = 225, EGFRm Phase 2	ORR 29.8% DOR 6.4 mo mPFS 5.5 mo mOS 11.9 mo
*Trop-2*	Datopotamab deruxtecan (Dato-DXd)	TROPION-PanTumor01 [45] *N* = 210 Phase 1	ORR 26% DOR 10.5 mo mPFS 6.9 mo mOS 11.4 mo
		TROPION-Lung02 [46] (Dato-DXd plus pembrolizumab doublet vs. Dato-DXd plus pembro plus Pt-CT) *N* = 20 vs. 42 Phase 1B	pembrolizumab vs. pembrolizumab plus CT: ORR 60% vs. 55% DCR 85% vs. 85%
		TROPION-Lung01 [47] (Dato-DXd vs. docetaxel) *N* = 299 vs. 305 Phase 3	Dato-DXd vs. docetaxel: ORR 26.4% vs. 12.8% DOR 7.1 mo vs. 5.6 mo mPFS 4.4 mo vs. 3.7 mo mOS 12.4 mo vs. 11 mo
		TROPION-Lung05 [48] *N* = 137, actionable mutations Phase 2	ORR 35.8% DOR 7 mo mOS 15.2 mo DCR 78.8%
	Sacituzumab govitecan (SG)	TROPiCS-03 [49] *N* = 54 Phase 1/2	ORR 16.7% DOR 6.0 mo mPFS 4.4 mo mOS 7.3 mo
	Sacituzumab tirumotecan SKB264	NCT04152499 [50] *N* = 43 Phase 2	EGFRm vs. EGFR-WT: ORR 60.0% vs. 26.3% mDOR 8.7 mo vs. 9.6 mo mPFS 11.5 mo vs. 5.3 mo mOS 22.7 mo vs. 14.1 mo
*c-Met*	Telisotuzumab vedotin (Teliso-V)	LUMINOSITY [51] *N* = 136 Phase 2	NSQ vs. SQ: ORR 36.5% vs. 11.1% DOR 6.9 mo vs. 4.4 mo
	ABBV-400	NCT05029882 [52] *N* = 57 Phase 1	ORR 43.8%
*CEACAM5*	Tusamitamab ravtansine	NCT02187848 [53] (high expressor ≥ 50% vs. moderate 1–50%) *N* = 64 vs. 28, NSQ Phase 1/2	High expressor: ORR 20.3% Moderate: ORR 7.1%
*B7-H3*	DS-7300	NCT04145622 [54] *N* = 5, SQ Phase 1/2	40% response DCR 80%

CEACAM5: cell adhesion molecule 5; HER2: human epidermal growth factor receptor 2; ADCs: antibody-drug conjugates; ORR: overall response rate; DOR: duration of response; mPFS: median progression-free survival; mOS: median overall survival; DCR: disease control rate; IHC: immunohistochemistry; EGFRm: EGFR-mutated; EGFR-WT: EGFR wild type; CT: chemotherapy; NSCLC: non-small cell lung cancer; NSQ: non-squamous; SQ: squamous; Trop-2: trophoblast cell-surface antigen 2; NE: not estimable

#### Anti-HER2 ADCs

Human epidermal growth factor receptor 2 (HER2) is an oncogene that drives cancer by promoting cell proliferation and inhibiting apoptosis. In NSCLC, HER2 overexpression incidence is 7.7–23% and is associated with poor prognosis, making it a promising target [55]. The first ADC approved for previously treated metastatic HER2-mutant NSCLC, trastuzumab deruxtecan, combines an anti-HER2 monoclonal antibody (trastuzumab) linked to a topoisomerase I inhibitor (deruxtecan). With a high DAR of 8, it delivers a cytotoxic payload while maintaining plasma stability. Its membrane permeable-payload enhances bystander killing effect, increasing efficacy in HER2 heterogeneous tumors [56]. In the phase 2 trial, DESTINY-Lung01, trastuzumab deruxtecan (6.4 mg/kg), patients had PFS of 8.2 months, OS of 17.8 months, and duration of response (DOR) was 9.3 months [36]. However, adverse events such as interstitial lung disease (ILD) or pneumonitis occurred in 26% of patients [36]. Ocular toxicities, such as blurred vision or visual impairment affected less than 5% of patients [57, 58]. Due to adverse events, the drug is being further studied in phase 3 study at a lower dosage of 5.4 mg/kg [59].

Other anti-HER2 ADCs, such as zanidatamab zovodotin (ZW49), a bispecific antibody directed against two non-overlapping HER2 epitopes linked to an anti-microtubule agent, showed an overall response rate (ORR) of 28% and disease control rate (DCR) of 72% across numerous cancer types (NSCLC rate not specified) in a phase 1 trial with a good safety profile, meriting future investigation [41]. Ado-trastuzumab emtansine (TDM1) is also an anti-HER2 ADC but was concluded to have limited efficacy for HER2-NSCLC [60].

#### Anti-HER3 ADCs

Although HER3 has poor tyrosine kinase activity on its own, it can form a heterodimer with HER2 or EGFR, promoting oncogenesis, metastasis, and drug resistance. It is overexpressed in 83% of NSCLC tumors, making it a valuable target. Patritumab deruxtecan (HER3-DXd), an ADC linking an anti-HER3 antibody to a topoisomerase I inhibitor, has a membrane-permeable payload that enhances its bystander killing effect and efficacy in heterogeneous tumors. In the phase 1 trial, HER3-DXd was evaluated in EGFR-mutated (EGFRm) NSCLC with prior EGFR TKI therapy. Among 57 patients, ORR was 39% and PFS was 8.2 months, demonstrating clinical activity regardless of EGFR related resistance mechanisms [42]. A phase 2 trial, HERTHENA-Lung01, also evaluated HER3-DXd in EGFRm patients (*n* = 225) previously treated with EGFR TKI and platinum-based chemotherapy. The ORR was 29.8%, DOR 6.4 months, PFS 5.5 months, and OS 11.9 months, which is promising [44]. Ongoing are a phase 3 trial, HERTHENA-Lung02, comparing HER3-DXd vs. platinum-chemotherapy in patients with disease progression after EGFR TKIs, and a phase 1 trial combining HER3-DXd and osimertinib in patients who have progressed on osimertinib [61, 62]. Treatment-related ILDs occurred in 5.3% of patients, and 1.8% experienced treatment-related AEs associated with death (pneumonitis, pneumonia, GI perforation, and respiratory failure) [44]. Overall, HER3-DXd was generally well-tolerated, with a low discontinuation rate (9%).

#### Anti-Trop-2 ADCs

Trophoblast cell-surface antigen 2 (Trop-2) plays a role in carcinogenesis, promoting self-renewal, proliferation, invasion, and survival. Trop-2 overexpression is associated with poor prognosis and metastasis, found in 60% of squamous cell carcinomas and 42–64% of adenocarcinomas [63].

Datopotamab deruxtecan (Dato-DXd) links a Trop-2 antibody with a topoisomerase I inhibitor. In the TROPION-PanTumor01 trial, which evaluated the drug in patients who progressed in 1 or 2 previous lines, Dato-DXd showed an ORR of 26% and PFS of 6.9 months, regardless of Trop-2 expression [45]. In NSQ patients and those with actionable mutations, the ORR increased to 35% with a DOR of 9.5 months [64]. The phase 3 TROPION-Lung01 reported an ORR of 26.4%, compared to 12.8% for docetaxel, with a PFS of 4.4 months and a DOR of 7.1 months, with particularly strong results in NSQ patients [47]. The phase 2 TROPION-Lung05 trial specifically showed elevated efficacy in patients with actionable mutations progressing beyond first line therapy: ORR of 35.8% and DOR of 7.0 months [48]. ILD occurred in 3.4% of patients on Dato-DXd, leading to one death. Other notable AEs were stomatitis, anemia, and increased amylase.

Sacituzumab govitecan (SG), another Trop-2 ADC, combines an anti-Trop-2 antibody with SN38, a topoisomerase inhibitor. The phase I/II IMMU-132 basket trial (TROPiCS-03) showed an ORR of 16.7%, DOR of 6.0 months, PFS of 4.4 months, and OS of 7.3 months. Similar to Dato-DXd, Trop-2 expression was not a predictor for clinical benefit [49]. However, a phase 3 trial, EVOKE-01, did not show SG had a significant OS improvement over docetaxel [65]. 9.8% of patients had AEs leading to treatment discontinuation, compared to 16.7% for docetaxel. Four treatment-related AEs led to death, with two involving neutropenia and two sepsis.

SKB264 (sacituzumab tirumotecan) is another Trop-2 ADC linked to a topoisomerase I inhibitor. The phase 2 expansion cohort study evaluated the drug in EGFRm and EGFR wild type (EGFR-WT) patients who had progressed on previous lines of therapy (median of three prior regimens) [50]. mPFS and median OS (mOS) were higher in EGFRm patients: mPFS was 11.5 months and 5.3 months, respectively, and mOS was 22.7 months and 14.1 months, respectively. Currently ongoing are a phase 3 global study in EGFRm NSCLC for third line and beyond treatment (NCT06074588) and phase 3 Chinese study in EGFRm NSCLC for second line treatment (NCT05870319) [66, 67].

#### Anti-c-MET ADCs

c-MET helps regulate cell proliferation, angiogenesis, survival, and cell motility. Its overexpression and amplification thus promotes tumor development. MET amplification is also a common drug resistance mechanism, particularly in EGFRm NSCLC [68]. Thus, ADCs targeting MET have been developed.

Telisotuzumab vedotin (Teliso-V) links the c-MET antibody with MMAE and was evaluated in the phase 2 trial, LUMINOSITY, in previously treated NSCLC patients with elevated c-MET expression (intermediate defined as 25–50%, high > 50%). The ORR was 36.5% in the NSQ EGFR-WT cohort, with 52.2% in the c-Met high subgroup and 24.1% in c-Met intermediate. The DOR was 6.9 months in NSQ c-MET high subgroup [51]. In contrast, ORR was modest in NSQ EGFRm and SQ patients. FDA granted Breakthrough Therapy Designation to Teliso-V for treatment of EGFR-WT NSQ NSCLC with high c-MET expression after disease progression [69]. Ongoing trials include a phase 3 study comparing Teliso-V with docetaxel in c-MET overexpressing NSCLC and a phase 1B study of Teliso-V as monotherapy or in combination with osimertinib, erlotinib, or nivolumab in c-MET overexpressed/EGFR-mutant metastatic NSCLC [70, 71].

ABBV-400, an anti-MET ADC linked to a topoisomerase I inhibitor, is in phase 1 testing for EGFR-WT NSQ NSCLC patients who progressed after platinum, immunotherapy, or targeted therapy [52]. It is showing ORR of 43.8% with PR in 43.8% of patients.

AZD9592 is a bispecific ADC targeting MET and EGFR linked to a topoisomerase I payload. The phase 1 trial, EGRET, is ongoing and aims to evaluate AZD9592 monotherapy and AZD9592 plus osimertinib in metastatic NSCLC with EGFRm (sensitizing L858R mutation or exon19 deletion) and EGFR WT64.

#### Other ADCs

Cell adhesion molecule 5 (CEACAM5) is overexpressed in 25% of NSCLCs and inhibits differentiation and apoptosis in cancer cells [72]. Tusamitamab ravtansine is an ADC targeting CEACAM5 linked to a cytotoxic maytansinoid, DM4. Phase 1 data showed, for NSQ high expressors (≥ 50% of tumor cells, *n* = 64) and moderate expressors (1–50%, *n* = 28), 13 had PR and 28 had SD, 2 had PR and 15 had SD, respectively [53]. A phase 3 trial also evaluated CEACAM5-DM4 ADC compared to docetaxel in NSQ NSCLC after failure of first-line treatment, but study endpoint was not reached and trial was discontinued [73].

B7-H3 is expressed in 80% of NSCLC cases and high levels were associated with poor OS [74]. DS-7300 is an ADC directed at B7-H3 linked with a topoisomerase I inhibitor. In phase 1/2 study, it was evaluated in heavily pre-treated patients (median 5 lines of previous treatment), resulting in ORR of 40%, albeit with low sample size (sqNSCLC *n* = 5) [54].

### Bispecific antibodies

**Figure 4:**
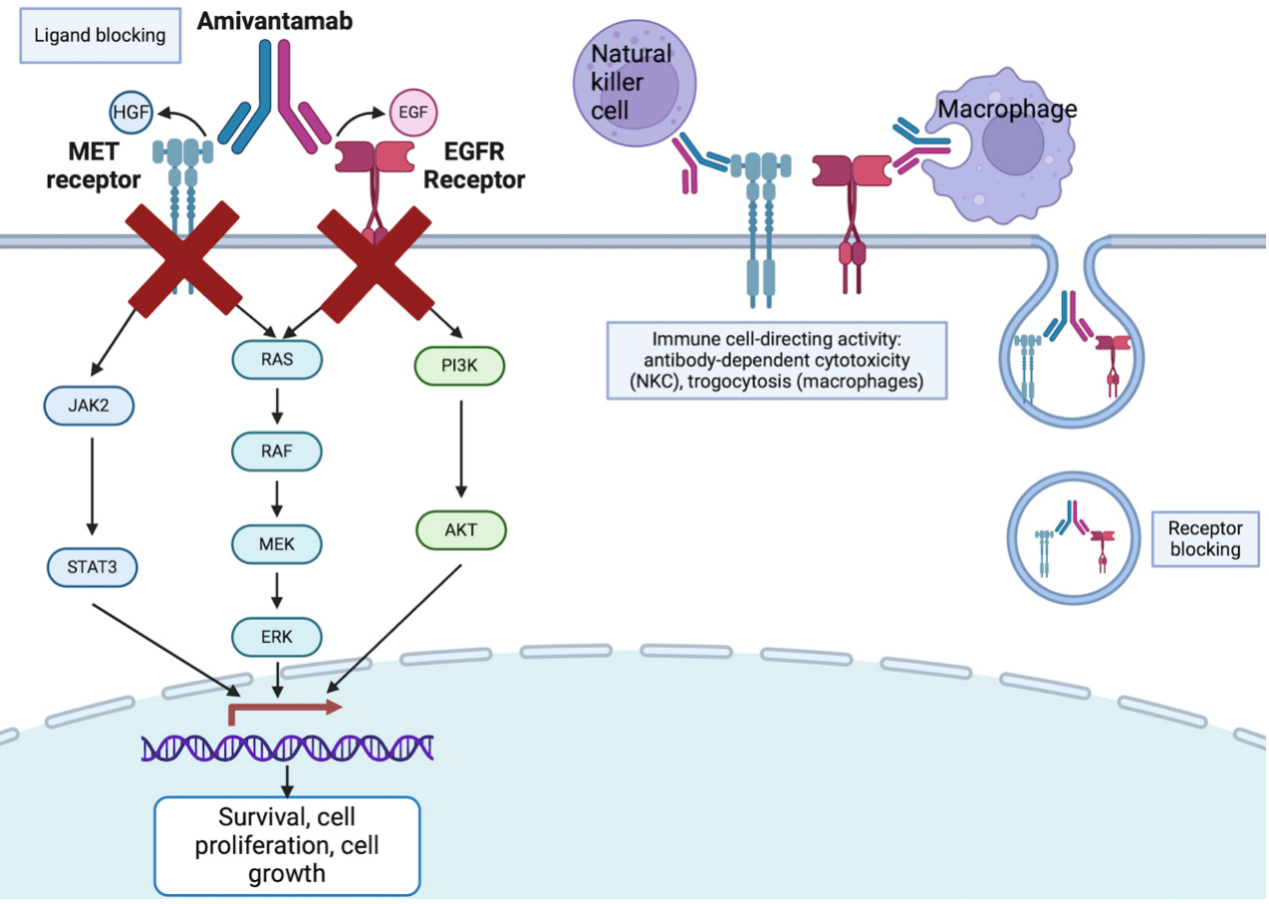
Mechanism of bispecific antibodies, with amivantamab as an example. Created in BioRender. Wang, K. (2024) BioRender.com/q78q582

Bispecific antibodies are recombinant molecules capable of simultaneously binding to two distinct antigens, enhancing their therapeutic potential (Figure 4) [75]. Bispecific antibodies are being explored in NSCLC to target both tumor-associated antigens and immune checkpoint molecules, aiming to overcome resistance mechanisms and enhance the efficacy of immune-based therapies (Table 2).

**Table 2:** Bispecific antibodies

**Target**	**Drug**	**Trial, phase**	**Outcomes**
*c-Met/EGFR*	Amivantamab	CHRYSALIS [68] *N* = 45, osimertinib-relapsed EGFRm Phase 1/1B	ORR 36% DOR 9.6 mo mPFS 4.9 mo
		MARIPOSA-2 [76] (CT vs. plus CT vs. plus lazertinib-CT) *N* = 263 vs. 131 vs. 263 Phase 3	CT alone vs. plus CT vs. plus Lazertinib-CT: ORR 36% vs. 64% vs. 63% DOR 5.6 vs. 6.9 vs. 9.4 mo mPFS 4.2 vs. 8.2 vs. 8.3 mo
*PD-L1/VEGF*	Ivonescimab (AK112)	NCT05184712 [77] (AK112 vs. CT) *N* = 161 vs. 161 Phase 3	AK112 vs. CT: mPFS 7.1 mo vs. 4.8 mo
*HER3/EGFR*	BL-B01D1	NCT05194982 [78] (EGFR-WT vs. EGFRm) *N* = 50 vs. 38 Phase 1 (ongoing)	EGFR-WT vs. EGFRm: ORR 44% vs. 63.2% mPFS 5.2 mo vs. 6.9 mo (immature) DCR 94% vs. 89.5%
	Izalontamab (SI-B001)	NCT05020457 [79] *N* = 45, SI-B001 plus doecetaxel Phase 2 (ongoing)	ORR 31.3% DCR 77.1%

ORR: overall response rate; DOR: duration of response; mPFS: median progression-free survival; DCR: disease control rate; EGFRm: EGFR-mutated; EGFR-WT: EGFR wild type; CT: chemotherapy; HER3: human epidermal growth factor receptor 3

Amivantamab is one such bispecific antibody (anti-MET and anti-EGFR) that enables three cytotoxic mechanisms: ligand blocking, receptor blocking, and immune cell-directing activity, through antibody dependent cellular cytotoxicity and trogocytosis. The phase 1/1B trial, CHRYSALIS, evaluated amivantamab as monotherapy, in combination with lazertinib (a brain-penetrant third generation EGFR TKI), or with carboplatin plus pemetrexed in post-osimertinib and post-platinum EGFRm NSCLC [68]. For the amivantamab-lazertinib arm, ORR was 36% and DOR was 9.6 months. Specifically for patients with disease progression after osimertinib and chemotherapy, ORR was 29% and median DOR was 8.6 months, which is promising. Another phase 3 trial, MARISOPA-2, compared chemotherapy, amivantamab-chemotherapy, and amivantamab-lazertinib-chemotherapy. Efficacy was increased in amivantamab-chemotherapy and amivantamab-lazertinib-chemotherapy cohorts compared to chemotherapy alone (ORR 64%, 63%, 36%, respectively; PFS 8.2 months, 8.3 months, 4.2 months) [76]. One significant amivantamab adverse effect is the high incidence of infusion side effect (≥ 20%) and a diverse range of skin side effect, most commonly acneiform eruption and pruritus [80, 81]. Potentially mitigating the high incidence of infusion side effects, subcutaneous amivantamab formulation (*n* = 206) was compared with traditional intravenous administration (*n* = 212), showing equal to superior effects: ORR was 30% and 33%, mPFS was 6.1 months and 4.3 months, respectively. Similarly, OS was significantly longer in subcutaneous group, with fewer infusion-related AEs (13% vs. 66%) and lower incidence of venous thromboembolism (9% vs. 14%) [82].

Ivonescimab (AK112 or SMT112) is a bispecific antibody targeting PD-L1 and VEGF; PD-L1 blockade prevents immune checkpoint from suppressing immune response while VEGF inhibition reduces angiogenesis. The phase 3 trial evaluated ivonescimab plus chemotherapy compared with chemotherapy alone in patients progressing on EGFR TKI treatment [77]. mPFS was higher in ivonescimab cohort at 7.1 months, compared to 4.8 month for chemotherapy. Subgroup analysis also demonstrated PFS benefit for patients treated with ivonescimab across almost all subgroups, including those with progression on third-generation EGFR TKIs and brain metastases. Similarly, ORR was higher for ivonescimab cohort (50.6% vs. 35.4%). However, grade ≥ 3 AEs were also more common in ivonescimab cohort (61.5% vs. 49.1%), including higher incidence of immune-related AEs and VEGF-related AEs.

BL-B01D1 is an anti-EGFR and anti-HER3 bispecific ADC linked to a topoisomerase I inhibitor. Based on first-in-human phase 1 data where they evaluated the drug in previously treated patients, EGFRm NSCLC cohort (*n* = 38) had ORR of 63.2%, DCR of 89.5% (*n* = 38), and EGFR-WT (*n* = 50) had 44.0% and 94.0%, respectively, demonstrating potential efficacy in both mutated and non-mutated pre-treated NSCLC tumors and in patients who have progressed on EGFR TKIs [78].

Izalontamab (SI-B001) is another EGFR and HER3 bispecific antibody. In the phase 2 trial, the drug was evaluated in EGFR/ALK-WT who failed first-line immunotherapy. The ORR was 31.3% with manageable toxicity, supporting continued investigation of SI-B001 plus docetaxel as second-line treatment [79].

### Additional emerging options

New forms of immunotherapy, like chimeric antigen receptor (CAR)-T cell therapy, T-cell receptor therapy (TCR) therapy, and mRNA vaccines, have generated interest among researchers and oncologists. Some promising drugs in NSCLC are shown in Table 3.

**Table 3:** Clinical trial data and ongoing trials testing novel treatments in NSCLC

**Type**	**Drug**	**Trial**	**Outcomes**	**Phase/No.**
CAR-T	EGFR-targeting CAR-T, modified by CXCR5	NCT05060796, NCT04153799		1 (*n* = 11), 1 (*n* = 11)
	EGFR-targeting CAR-T, knocked-out TGFβ receptor	NCT04976218		1 (*n* = 30)
	MSLN-targeting CAR-T plus anti-PD-1 cells (for mesothelioma)	NCT04577326		1 (*n* = 30)
	MSLN-targeting CAR-T	NCT04489862		1 (*n* = 10)
	PSCA/MUC1/TGFβ/GPC3-targeting CAR-T	NCT03198052		1 (*n* = 30)
	MUC1-targeting CAR-T plus anti-PD-1 cells	NCT03525782		1/2 (*n* = 60)
	MUC1-targeting CAR-T, 3 + 3 dose design	NCT05239143		1 (*n* = 100)
TCR	KK-LC1-targeting TCR, followed by high dose IL-2	NCT05483491, NCT03778814		1 (*n* = 42), 1 (*n* = 30)
	MAGE A4/8-targeting TCR	NCT03247309		1 (*n* = 7)
	MAGE A10-targeting TCR	NCT02592577 [83]	11% PR 36% SD	1 (*n* = 11)
	Neoantigen-targeting TCR (NSCLC not specified)	NCT03412877		2 (*n* = 270 total)
	NY-ESO-1 targeting TCR, followed by low dose IL-2	NCT02457650 [84]	25% PR 25% SD	1 (*n* = 4)
	NY-ESO-1 targeting TCR	NCT05296564		1 (*n* = 3)
	KRAS, EGFR, TP53 targeting TCR; followed by IL-2 (NSCLC not specified)	NCT05194735		1/2 (*n* = 180 total)
mRNA	CEA-targeting RNA-pulsed DCs (NSCLC not specified)	NCT00004604 [85]	4% CR 8% PR 12% SD	1 (*n* = 24)
	SOCS1, MUC1, survivin-targeting RNAs	NCT02688686		1/2 (*n* = 30)
	KRAS-targeting RNA	NCT03948763, NCT05202561		1 (*n* = 70), 1 (*n* = 10)
	﻿NY-ESO-1, MAGEC1/C2, survivin, trophoblast-targeting RNAs (CV9201)	NCT00923312 [86]	31% SD 69% PD mPFS 5.0 mo mOS 10.8 mo	1/2 (*n* = 46)
	NY-ESO-1, MAGEC1/C2, MUC1, survivin, 5T4 RNAs (CV9202 or BI1361849)	NCT03164772 [87] (Arm A: mRNA plus durvalumab; Arm B mRNA plus durvalumab plus tremelimumab)	Arm A: ORR 29% Arm B: ORR 11%	1/2 (*n* = 62)
		NCT01915524 [88]	4% PR (with pemetrexed) 46.2% SD	1 (*n* = 26) Terminate
	Personalized neoantigen mRNA	NCT03908671		1 (*n* = 24)

CAR: chimeric antigen receptor; TCR: T-cell receptor therapy; ORR: overall response rate; mPFS: median progression-free survival; mOS: median overall survival; CR: complete response; PR: partial response; SD: stable disease; PD: progressive disease; GPC3: phosphatidylinositol proteoglycan 3; MUC1: mucin 1; NSCLC: non-small cell lung cancer; PSCA: prostate stem cell antigen

#### CAR-T

Clinically, CAR-T cell therapy has shown significant success in treating hematological malignancies, with an overall remission rate of 80%. Recent progress suggests that CAR-T-cell therapy is also a promising strategy for treating solid tumors like NSCLC [89, 90]. CAR-T cells are T-cells that are genetically engineered outside of the patient’s body to express antibodies that bind to antigens expressed on cancer cells. They consist of three components: a domain that binds to antigens, a signaling domain that activates the T-cell, and a hinge connecting the two. CAR-Ts, now in their fifth generation, have significantly improved, especially in the signaling domain that triggers T-cell activation.

EGFR transduces extracellular signaling for cell growth. Around 15% of NSCLCs express EGFR, making it an important target for CAR-T therapy. A preclinical study evaluated the efficacy of CAR-T targeting EGFR in mouse models. Metastasis of cancer in the mice was inhibited by the CAR-T therapy and survival was prolonged with no serious side effects [91]. A phase 1 trial is evaluating the efficacy of CAR-T cells targeting EGFR in patients with advanced-stage NSCLC (NCT05060796), where the therapy was mostly well-received except for a significant increase in serum lipase levels graded 3–4. A recent study also investigated intraventricular EGFR-directed CAR-T cell therapy in patients (*n* = 3) with recurrent glioblastoma [92]. They found rapid tumor regression on radiographic imaging; however, the effects were transient. More research needs to be done on the longevity of therapeutic effects and dosing to limit side effects.

Mesothelin (MSLN) is also abundantly expressed in NSCLC and is correlated with poor prognosis and chemotherapy resistance. Despite the potential of the target, clinical trials to date for CAR-T targeting MSLN as monotherapy have not been effective. The phase 1 study evaluating MSLN CAR-T cells was halted due to disease progression in many patients. Additionally, there were six serious AEs and one fatality associated with the highest dosage of CAR-T cells (NCT02414269). However, other clinical trials have shown that combining MSLN CAR-T with standard therapy may increase efficacy [93]. One study is evaluating the safety of CAR-T cells plus an anti-PD-1 agent in treating mesothelioma (NCT04577326).

Prostate stem cell antigen (PSCA) is frequently amplified in NSCLC, but its function is cell type and context-dependent. Third-generation CAR-T targeting PSCA was shown to postpone the progression of NSCLC in patient-derived xenograft models. Combination therapy with mucin 1 (MUC1)-targeted CAR-T cells resulted in compounded effect, substantially reducing tumor size more than monotherapy [94]. One clinical trial is currently evaluating PSCA CAR-T [along with MUC1, TGFβ, and phosphatidylinositol proteoglycan 3 (GPC3) in advanced NSCLC (NCT03198052)].

MUC1 becomes notably expressed following cancerous changes, especially in lung adenocarcinoma. A preclinical study showed that MUC28z CAR-T cells, designed to target MUC1, were effective in recognizing and attacking MUC1-positive tumors in mice, significantly reducing tumor size within four days with a prolonged ability to attack cancer cells for up to 81 days after treatment [95]. An in-human pilot clinical study combined MUC1 CAR-T cells with PD-1 knockout cells, showing promise in shrinking primary tumors in advanced NSCLC, although it was less effective against metastases (NCT03525782).

#### T-cell receptor therapy

TCR therapy is when a patient’s T-cells are collected and engineered to express a TCR that targets a specific tumor-specific or tumor-associated antigen. The targets for these TCRs are antigens unique to cancer cells and minimally expressed in normal cells. A significant benefit of TCR-T cells in treating solid tumors is their ability to recognize antigens located inside the cell, as well as those on the cell surface, in contrast to CAR-T cells which are limited to surface antigens. Moreover, TCR-T cells can be activated by much lower levels of antigen than CAR-T cells, requiring only a few epitopes per cell to be effective. However, for these TCR-T cells to work, patients must not only have the tumor antigen but also the corresponding HLA allele that presents this antigen [96]. Real-time technical advancements in neoantigen multitargeting have significantly improved the availability of TCR-T therapy for patient use, particularly through breakthroughs in rapid whole-exome sequencing and RNA sequencing, allowing for the identification of patient-specific neoantigens.

TCR-T [97] therapy in NSCLC research is still at an early stage, with most clinical trials showing good tolerability but limited efficacy. For example, in a phase 1 trial, out of four patients treated with New York esophageal squamous cell carcinoma (NY-ESO-1) TCR-T cells, one showed partial tumor shrinkage, one had SD, and none experienced severe side effects [84]. Another study evaluating MAGE-A10-specific-TCR-T cells showed acceptable safety profile, with some NSCLC patients experiencing cytokine-release syndrome, and had promising results, with 11% PR and 36% SD (*n* = 11) [83]. In another phase 1/2 trial evaluating mesothelin-targeting TCR therapy in mesothelioma, NSCLC, ovarian cancer, or cholangiocarcinoma, 20% responded to the treatment, 77% experienced some level of disease control, and 70% survived at least six months (*n* = 30), with high-grade pneumonitis and cytokine release syndrome (CRS) observed in 16% and 25%, respectively [98]. Further development in TCR therapy depends on the identification and targeting of new antigens. One challenge is that engineered TCRs are HLA restricted and require HLA matching to be effective. Most therapies are designed to target HLA-A*0201, which is common in Caucasians but less common among African and Asian populations [99].

#### mRNA cancer vaccines

The goal of mRNA vaccines is to activate an immune response against cancer by targeting tumor antigens. Modern mRNA vaccines typically encode the CEA, PSA, MAGE1, survivin, tyrosinase, hTERT, or WT1 antigens, but there has been an increasing shift towards personalized neoantigen-based vaccines [100]. This approach tailors vaccines to individual patients, potentially overcoming the limitations of traditional antigen targets and offering increased efficacy. Other advancements in mRNA cancer vaccines have focused on overcoming the molecule’s inherent instability and enhancing immunogenicity through modifications such as nucleoside changes, adjustments to the 5’ and 3’ untranslated regions, and improvements to the poly(A) tail and 5’ cap structure [101, 102]. Additionally, lipid nanoparticle (LNP) delivery systems have shown increased clinical efficacy, offering advantages such as high immunogenicity, ease of manufacturing, and effective cellular entry, compared to the more complex dendritic cell-based platforms of earlier studies [103].

CV9201 is a mRNA vaccine targeting NY-ESO-1, MAGEC1/C2, survivin, and trophoblast glycoprotein. Despite being well-tolerated, with mild AEs, the phase 1 trial in patients following SD post-first-line treatment (*n* = 46) demonstrated infrequent and not durable immune responses [86]. Thus, a similar vaccine (CV9202 or BI1361849) was synthesized to target NY-ESO-1, MAGEC1/C2, survivin, MUC1, and 5T4. In a phase 1 trial, it was evaluated in combination with radiation therapy for stage IV NSCLC after first-line chemotherapy or TKI treatment [88]. Results showed good tolerance and an increase in antigen-specific immune responses. However, only one patient (*n* = 26) achieved PR, although six patients experienced shrinkage of non-irradiated lesions that did not qualify as PR. Another phase 1/2 study assessed CV9202 in combination with ICIs (non-randomized) [104, 105]. Arm A received mRNA plus durvalumab (*n* = 24) and Arm B mRNA plus durvalumab and tremelimumab (*n* = 37). Arm A had ORR of 29% and 71% DCR, while Arm B had 11% ORR and 53% DCR. Arm A was 26.3% PR, 36.8% SD, 36.8% PD; Arm B 11.1%, 29.6%, 59.3%, respectively. Combining durvalumab with mRNA vaccine yielded better treatment than durvalumab monotherapy or combination with chemotherapy, suggesting further research to be done about combining vaccines and ICIs.

Another mRNA target is KRAS, which is frequently overexpressed in NSCLC. A phase 1 study is studying mRNA-5671/V941, a nanoparticle-encapsulated mRNA vaccine (NCT03948763). This trial is evaluating the vaccine’s efficacy for patients with KRAS-mutant NSCLC, colorectal cancer, and pancreatic adenocarcinoma, either as a standalone treatment or alongside pembrolizumab. Another trial (NCT05202561) is recruiting patients with advanced KRAS-mutated NSCLC to evaluate mRNA vaccine safety, tolerability, anti-tumor efficacy, immunoreactivity, and pharmacokinetics either as monotherapy or in combination with ICIs.

Another trial is assessing a customized neoantigen mRNA vaccine as monotherapy in advanced stages of NSCLC and esophageal cancer after failure of standard treatment. The goal is to investigate the safety and to conduct an initial evaluation of the vaccine’s effectiveness. Trial is actively enrolling (NCT03908671).

## Conclusions

The review has provided a narrative discussion of current and future treatment approaches for second line therapy in NSCLC. Further improvements have been seen in the detection of progression in NSCLC through the evolution of molecular testing techniques, particularly in patients with *EGFR* mutations and *ALK* rearrangements [106]. Regular monitoring through repeat molecular testing can help identify resistance mutations early, enabling timely adjustments to therapeutic strategies. Liquid biopsies are recommended to evaluate circulating tumor DNA (ctDNA), allowing for minimally invasive, real-time monitoring of tumor evolution and the emergence of resistance mutations. This approach has been especially useful in detecting T790M mutations in patients undergoing EGFR-TKI therapy, allowing for the switch to third-generation TKIs like osimertinib, which target resistant mutations more effectively. Droplet-digital PCR has also been shown to offer high sensitivity and specificity for detecting low-abundance T790M mutations [107]. Additionally, new applications for ctDNA, such as monitoring real-time therapy response, minimal residual disease (MRD) testing, and its potential as a predictive biomarker for immunotherapy, are under investigation [108, 109]. In cases where EGFR T790M is not detected, testing for alternative resistance mechanisms, such as MET or ERBB2 amplification, may guide further therapeutic strategies [110].

The optimization of response to ADCs, especially Trop-2 targeting ADCs, necessitates the development of more robust biomarkers [111]. Current reliance on biomarkers like PD-L1 and, occasionally, tumor mutational burden (TMB) provides an incomplete picture of the tumor microenvironment [112]. Additionally, challenges such as tumor heterogeneity and the accuracy of sampling biopsies present problems [113]. Currently, several potential biomarkers are being studied to better predict response to Trop-2 ADCs. For instance, Trop-2 expression itself as a biomarker is under investigation, although early studies have shown that ADC response can occur regardless of Trop-2 expression levels [114]. A novel biomarker measuring Trop-2 normalized membrane ratio by quantitative continuous scoring (QCS-NMR) was found to be predictive of better outcomes in patients treated with Dato-DXd, a Trop-2 ADC. Patients with Trop-2 QCS-NMR+ tumors had a higher ORR of 32.7% and PFS of 6.9 months treated with Dato-DXd compared with 10.3% ORR and 4.1 months PFS for docetaxel. In contrast, the Trop-2 QCS-NMR– group showed no significant advantage for Dato-DXd over docetaxel in terms of ORR or PFS [115]. Additionally, biomarkers related to DNA damage response pathways, such as BRCA1/2 mutations, may help predict sensitivity to Trop-2 ADCs linked to topoisomerase inhibitors, which rely on the disruption of DNA repair mechanisms [114]. Moreover, further exploration of the role of immune cell infiltration, such as leukocyte infiltration, which has already been recognized as a prognostic and predictive biomarker in breast cancer, could also offer insights into which NSCLC tumors are more likely to respond to Trop-2 ADCs [113].

The sequencing of therapies, particularly in the context of adoptive cell therapies, is critical to maximizing therapeutic efficacy and minimizing toxicities. The order in which these therapies are administered can influence both their effectiveness and the patient's ability to tolerate them [116]. Adoptive cell therapies, such as CAR-T and TCR-T cells, often come with significant toxicities, including CRS and neurotoxicity [117]. These adverse effects can limit the feasibility of subsequent treatments and impact overall treatment strategy. Understanding the cumulative and overlapping toxicities of different therapies is essential for developing optimal sequencing protocols. For example, administering low toxicity treatments initially may preserve the patient’s health status, making them better candidates for more aggressive therapies later. Pre-emptive strategies to mitigate these toxicities, such as the use of IL-6 inhibitors for managing CRS or corticosteroids for neurotoxicity, can also play a crucial role in managing side effects [118, 119]. In addition, comprehensive management protocols, including supportive care measures and regular monitoring for early signs of toxicity, can further mitigate the adverse effects of these therapies. For instance, monitoring cytokine levels can help predict the onset of CRS, allowing for timely intervention [120]. The use of biomarkers to predict and monitor toxicities can also guide therapy adjustments, ensuring that patients receive the most effective treatment with the least harm [113].

In the evolving landscape of immunotherapies for NSCLC, understanding the tumor microenvironment, including the roles of neutrophils and macrophages may add depth to therapeutic strategies. Neutrophils are the dominant component of immune cells composition in NSCLC, existing in the N1 anti-tumor and N2 pro-tumor state [121]. N2 tumor-associated neutrophils have emerged as significant immunosuppressive factors. Smad3 signaling plays a key role in maintaining this pro-tumor N2 state. Inhibiting Smad3 shifts neutrophils toward the anti-tumor N1 phenotype, enhancing tumor regression and suggesting Smad3 as a promising therapeutic target for cancer immunotherapy. Another strategy for neutrophil-based cancer therapies involves utilizing neutrophils and their derivatives or mimetics as therapeutic agents and drug delivery systems [122]. Other strategies focus on inhibiting the pro-tumor functions of neutrophils while simultaneously enhancing their anti-tumor properties [122]. Similarly, macrophages and processes like macrophage-myofibroblast transition (MMT) are potential novel targets. MMT generates a subset of cancer-associated fibroblasts, which play an underdefined role in tumor microenvironment driven cancer progression. Similar to neutrophils, Smad3 has been identified as a regulator for the transition; targeting Smad3 offers a potential therapeutic strategy to suppress cancer progression [123]. Mincle is also crucial for tumor-associated macrophage-driven cancer progression. An animal model study has utilized RNA interference and ultrasound-microbubble-mediated gene transfer (USMB) to develop gene therapy to silence Mincle, which successfully inhibited tumor progression and promoted the anticancer M1 phenotype of TAMs in lung carcinoma and melanoma xenografts [124].

The path forward necessitates further inquiry to elucidate the therapeutic efficacy and safety profiles of new therapeutics. Concurrently, translational research endeavors must focus on optimizing drug delivery mechanisms, developing toxicity management strategies, identifying novel protein targets for antibody engagement, and investigating the potential of immunotherapy-chemotherapy combinations. Furthermore, the exploration of cutting-edge topics such as molecular testing to detect progression, more effective biomarkers, and drug sequencing represents a pivotal frontier in NSCLC treatment. This multifaceted approach, integrating groundbreaking drug development with the nuances of patient-specific therapeutic needs, is paramount for advancing NSCLC treatment.

## Abbreviations

ADCs: antibody-drug conjugates
CAR: chimeric antigen receptor
CEACAM5: cell adhesion molecule 5
CR: complete response
CRS: cytokine release syndrome
DAR: drug-antibody ratio
Dato-DXd: datopotamab deruxtecan
DCR: disease control rate
DOR: duration of response
EGFRm: EGFR-mutated
EGFR-WT: EGFR wild type
GPC3: phosphatidylinositol proteoglycan 3
HER2: human epidermal growth factor receptor 2
ICIs: immune checkpoint inhibitors
ILD: interstitial lung disease
mOS: median overall survival
mPFS: median progression-free survival
MUC1: mucin 1
NSCLC: non-small cell lung cancer
NSQ: non-squamous
ORR: overall response rate
OS: overall survival
PD: progressive disease
PFS: progression-free survival
PR: partial response
PSCA: prostate stem cell antigen
SBRT: stereotactic body radiation therapy
SD: stable disease
SG: sacituzumab govitecan
SOC: standard of care
SQ: squamous
TCR: T-cell receptor therapy
TDM1: ado-trastuzumab emtansine
Teliso-V: telisotuzumab vedotin
TKI: tyrosinase kinase inhibitor
Trop-2: trophoblast cell-surface antigen 2

## Declarations

### Copyright

© The Author(s) 2024.
